# Supplementary material for: Transformation of Residual Açai Fruit (Euterpe oleracea) Seeds into Porous Adsorbent for Efficient Removal of 2,4-Dichlorophenoxyacetic Acid Herbicide from Waters
Source: Molecules. 2022 Nov 11;27(22):7781. doi: 10.3390/molecules27227781 (PMC9695194; doi:10.3390/molecules27227781)
Supplement: Supplementary file 1 [file molecules-27-07781-s001.zip › molecules-1979450-supplementary.pdf]

Supplementary material

S1. Characterization techniques

Fourier-transform infrared spectroscopy (FT-IR) was performed by an IR-Prestige-21 (Shimadzu) equipment to obtain information regarding the chemical structure of the raw and modified materials. In this step, 50 mg of each material was pressed separately with 50 mg of dry potassium bromide. The spectra were obtained with a resolution of 4 cm^{-1}, at several sequential scans in the range of 4500-500 cm^{-1}. The respective analyzes were carried out at room temperature (298 K) in a dehumidified room. Scanning electron microscopy (SEM) analysis was performed on a Vega 3 SB (Tescan) apparatus, with a working voltage of 10 kV, and image magnification of 500x, 1000x, and 3000x, to investigate the surface morphology of the materials. X-ray diffraction (XRD) analysis was performed with the aid of a computer-controlled X-ray diffractometer (Miniflex 300, Rigaku) to identify the crystallographic structure. The N_2 adsorption/desorption isotherms were performed using an ASAP 2020 (Micrometrics) apparatus. The material's specific surface area (SBET, m^2 g-1) was estimated based on the Brunauer-Emmett-Teller (BET) theory. The total pore volume (V_T, cm^3 g^{-1}) was determined considering the amount of N_2 adsorbed on the material surface at a relative pressure (P/P_0) of 0.99. The total pore volume was determined considering the N_2 volume adsorbed at P/P_0 values of 0.1 and 0.95. Scanning electron microscopy (SEM) coupled with energy dispersive spectroscopy (EDS) (Vega 3 SB, Tescan) was used to obtain the material surface image and its atomic composition.

S2. Batch adsorption equations

The adsorption capacity at any time (q_t, mg g^{-1}), adsorption capacity at the equilibrium (q_e, mg g^{-1}), and removal percentage (R, %) values were calculated by the Equations (1), (2) and (3), respectively:

$$q_t = (C_0 – C_t)\ \frac{V}{m} \tag{1}$$

$$q_e = (C_0 – C_e)\ \frac{V}{m} \tag{2}$$

$$R = \frac{(C_0 – C_t)}{C_0}\ 100 \tag{3}$$

Where: C_0 is the initial adsorbate concentration in the liquid phase (mg $L^{–1}$), C_e is the equilibrium concentration of the adsorbate in the liquid phase (mg $L^{–1}$), C_t is the adsorbate concentration in the liquid phase at any time (mg $L^{–1}$), m is the mass of adsorbent (g), and V is the volume of solution (L).

S3. Isotherm models

The Langmuir model (Eq. 4), Freundlich model (Eq. 5), and Sips (Eq. 6) are presented as follows:

$$q_e = q_L\ \frac{K_L C_e}{1+K_L C_e} \tag{4}$$

$$q_e = K_F C_e^{1/n_f} \tag{5}$$

$$q_e = q_{mS}\ \frac{K_S C_e^{nS}}{1+K_S C_e^{nS}} \tag{6}$$

Where: q_L is the maximum adsorption capacity of Langmuir model (mg g^{-1}), K_L is the Langmuir constant (L mg^{-1}); K_F is the Freundlich constant ((mg $g^{–1}$) (mg $L^{–1})^{–1/n}$); $1/n_f$ (dimensionless) is the heterogeneity factor; q_{mS} is the maximum adsorption capacity (mg g^{-1}), K_S is the Sips constant $(L\ mg^{-1})^{nS}$, n_S is the Sips exponent (dimensionless and $0 \geq n_S \leq 1$)

S4. Thermodynamics estimation

The Gibbs free energy (ΔG^0, kJ mol^{-1}), enthalpy change (ΔH^0, kJ mol^{-1}), and entropy change (ΔS^0, kJ mol^{-1} K^{-1}) were calculated by the following Equations:

$$K_e = \sqrt[n_s]{K_S} M_W \qquad (7)$$

$$\Delta G^0 = -RT\ln(K_e) \qquad (8)$$

$$\Delta G^0 = \Delta H^0 - T\Delta S^0 \qquad (9)$$

$$\ln(K_e) = \frac{\Delta S^0}{R} - \frac{\Delta H^0}{RT} \qquad (10)$$

Where: K_e is the equilibrium constant (dimensionless); K_S is the Sips constant ((mg g^{-1}) (mg $L^{-1})^{-1/ns}$, n_S is the Sips exponent (dimensionless and $0 \geq n_S \leq 1$), T is the temperature (K); R is the universal gas constant (8.31×10^{-3} kJ mol^{-1} K^{-1}).

S5. LDF model

For describing the adsorption kinetics, the linear driving force model (LDF) was selected, where the adsorption rate can be expressed as:

$$\frac{dq_t}{dt} = k_{LDF}\left(q^* - q_t\right) \qquad (11)$$

$$q_t\ (t=0)=0 \qquad (11.a)$$

where: q_t is the adsorption capacity at any time (mg g^{-1}), q^* is the adsorption capacity at the equilibrium (mg g^{-1}), k_{LDF} is the LDF mass transfer coefficient (min^{-1}). The solution of the model depends on the best-fitted isotherm, and in this case, the Sips model was chosen:

$$q^* = q_{mS}\frac{K_S C_e^{ns}}{1+K_S C_e^{ns}} \qquad (12)$$

The equilibrium concentration can be correlated with the adsorption capacity through the mass balance equation:

$$C_e = C_0 - D_0\bar{q} \qquad (13)$$

Where C_0 is the initial concentration (mg L^{-1}), D_0 is the adsorbent dosage (g L^{-1}).

Substituting Eqs. (12) and (13) into (11), results:

$$\frac{d\bar{q}}{dt}=k_{LDF}\left(q_{mS}\frac{K_S(C_0-D_0\bar{q})^{ns}}{1+K_S(C_0-D_0\bar{q})^{ns}}-\bar{q}\right) \quad (14)$$

The diffusivity for a homogeneous particle can be estimated from the LDF kinetic lumped parameter:

$$D_S=\frac{R_p{}^2 k_{LDF}}{15} \quad (15)$$

R_p is the adsorbent particle radius, and D_S is the surface diffusivity ($cm^2\ s^{-1}$).

S6. Statistical evaluation

The determination coefficient (R^2) (Eq. 16), adjusted determination coefficient (R^2_{adj}) (Eq. 17), average relative error (ARE) (Eq. 18), and mean square error (MSR) (Eq. 19) are given by the following Equations:

$$R^2 = 1-\frac{\sum_{i=1}^{n}\left(y_{exp}-y_{pred}\right)^2}{\sum_{i=1}^{n}\left(y_{exp}-\bar{y}_{exp}\right)^2} \quad (16)$$

$$R^2_{adj} = 1-\left(1-R^2\right)\frac{(n-1)}{(n-p-1)} \quad (17)$$

$$ARE = \frac{100\%}{n}\sum_{i=1}^{n}\left|\frac{y_{exp}-y_{pred}}{y_{exp}}\right| \quad (18)$$

$$MSR = \frac{1}{n-p}\sum_{i=1}^{n}\left(y_{exp}-y_{pred}\right)^2 \quad (19)$$

where: y_{exp} is the experimental value; y_{pred} is the predicted value; n is the number of experimental values; p is the number of parameters according to the model.
